# Clinical characteristics of advanced non-small cell lung cancer patients with *EGFR* exon 20 insertions

**DOI:** 10.1038/s41598-021-98275-3

**Published:** 2021-09-21

**Authors:** Chie Morita, Tatsuya Yoshida, Masayuki Shirasawa, Ken Masuda, Yuji Matsumoto, Yuki Shinno, Shigehiro Yagishita, Yusuke Okuma, Yasushi Goto, Hidehito Horinouchi, Noboru Yamamoto, Noriko Motoi, Yasushi Yatabe, Yuichiro Ohe

**Affiliations:** 1 Department of Thoracic Oncology, National Cancer Center Hospital, 5-1-1 Tsukiji, Chuo-ku, Tokyo, 104-0045 Japan; 2 Division of Molecular Pharmacology, National Cancer Center Research Institute, Tokyo, Japan; 3 Department of Diagnostic Pathology, National Cancer Center Hospital, Tokyo, Japan

**Keywords:** Non-small-cell lung cancer, Lung cancer

## Abstract

Epidermal growth factor receptor (*EGFR*) exon 20 insertion mutations (Exon20ins) account for 4–12% of all *EGFR* mutations in non-small cell lung cancer (NSCLC) patients. Data on the differences in clinical characteristics between patients with Exon20ins and major mutations (M-mut) such as exon 19 deletion and L858R are limited. We retrospectively reviewed advanced NSCLC patients with *EGFR* mutations, who were treated with systemic therapy between January 2011 and December 2019. We identified 23 patients with Exon20ins and 534 patients with M-mut. In Exon20ins patients, the median age was 60 (range 27–88) years, and females and never-smokers were predominant. Clinical characteristics were similar in the two groups. In Exon20ins patients, 17 patients received platinum doublet as first-line therapy, and the overall response rate (ORR) and median progression-free survival (mPFS) were 11.8% and 8.9 months. Additionally, seven patients received conventional EGFR-tyrosine kinase inhibitors (TKIs), and eight patients anti-PD-1 antibodies in any-line therapy. ORR and mPFS of EGFR-TKIs and anti-PD-1 antibodies were 0%, 2.2 months and 25%, 3.1 months, respectively. Overall survival was significantly shorter in Exon20ins patients than in M-mut patients (29.3 vs. 43.4 months, p = 0.04). The clinical outcomes in Exon20ins patients were not satisfactory compared to M-mut patients.

## Introduction

Epidermal growth factor receptor (*EGFR*) mutations mainly occur between exons 18 and 21 in non-small cell lung cancer (NSCLC), and are commonly found in never smokers, women, and patients with lung adenocarcinoma^1,2^. The frequency of *EGFR* mutations has been reported to be 47.9% in adenocarcinoma and 4.6% in lung squamous cell carcinoma among East Asian populations, and 19.2% in lung adenocarcinoma and 3.3% in lung squamous cell carcinoma among Western populations^3^. The most common genetic mutation is the deletion of exon 19 and L858R in exon 21, which accounts for about 70–80% of all *EGFR* mutations^4,5^. Most advanced NSCLC patients with these *EGFR* mutations respond to treatment with EGFR-tyrosine kinase inhibitors (EGFR-TKIs) such as gefitinib, erlotinib, afatinib, and osimertinib, with median progression-free survivals (mPFS) of 9.2–18.9 months^6–11^.

Exon 20 insertion mutations are the third most common subtype of *EGFR* mutation, which accounts for about 4–12% of all *EGFR* mutations, and are mutually exclusive with other known driver mutations. Exon 20 insertion mutations are also associated with a lack of sensitivity to the aforementioned EGFR-TKIs^4,12–14^. The standard treatment for patients with exon 20 insertion is systemic chemotherapy, which is similar to the treatment of other NSCLC cases without driver mutations^15,16^. On the other hands, novel targeted therapies against NSCLC with *EGFR* exon 20 insertion mutations, such as poziotinib^17^, mobocertinib (TAK-788)^18,19^, and amivantamab (JNJ-61186372)^20^ have been developed in preclinical and early clinical trials. There has been a growing interest on this subgroup of *EGFR*-mutant NSCLC patients.

Few studies have focused on the differences in clinical characteristics between patients with *EGFR* exon 20 insertions and major mutations. Our study therefore aimed to clarify the clinical characteristics and outcomes, including the efficacy of systemic treatment in patients with *EGFR* exon 20 insertion mutations, compared with those with major mutations.

## Patient and methods

### Subjects

We retrospectively reviewed advanced NSCLC patients with *EGFR* exon 20 insertion mutations treated with systemic chemotherapy, and those with *EGFR* major mutations (e.g., deletion in exon 19 and L858R in exon 21) treated with EGFR-TKIs as initial treatment at the National Cancer Center Hospital in Japan between January 2011 and December 2019. We collected data on patient characteristics, variants of exon 20 insertion, and clinical outcomes from medical records.

### Detection of EGFR mutation including exon 20 insertion mutations

The diagnosis of *EGFR* mutation including exon 20 insertion was performed based on PCR-based methods (therascreen EGFR RGQ PCR Kit [Scorpion-ARMS technology]; QIAGEN, Hilden, Germany, and Cobas E*GFR* Mutation Test v2; Roche Diagnostics, Basel, Switzerland)^21,22^ and next-generation sequencing (NGS) testing (OncoGuide NCC Oncopanel System, Sysmex, Kobe, Japan)^23^.

### Statistical analysis

To evaluate the differences in clinical characteristics between the patients, Fisher’s exact test was performed. The treatment effect was evaluated based on the Response Evaluation Criteria in Solid Tumors (RECIST version 1.1)^24^. The overall response rate (ORR) was defined as the percentage of patients with the best overall response of complete response (CR) or partial response (PR). We also used the Kaplan–Meier method to investigate PFS and overall survival (OS). OS was defined as the time from the date of diagnosis of advanced disease to death. PFS was defined as the time from the start of treatment to disease progression or death and was censored on the date the patient was last known as progression-free. All statistical analyses were performed using the EZR ver. 1.41^25^. This study was approved by the Ethics Committee of the National Cancer Center Hospital (2015-355 and 2019-123).

### Ethics approval

This study was performed in line with the principles of the Declaration of Helsinki. Approval was granted by the Ethics Committee of National Cancer Center Hospital in Japan (2015-355 and 2019-123).

### Consent to participate

Informed consent was obtained from all individual participants included in the study.

### Consent for publication

Patients has consented regarding publishing their data.

## Results

### Patient characteristics

We identified 23 patients with exon 20 insertions and 534 patients with major mutations, including 285 patients with an exon 19 deletion and 249 patients with an L858R mutation in exon 21. Patient characteristics according to *EGFR* mutation status are shown in Table 1. Patients with exon 20 insertions were significantly younger than those with major mutations (median age 60 vs. 66 years, p = 0.017). There were no significant differences in baseline characteristics between patients with exon 20 insertions and major mutations, except for age. Regarding the metastatic spread, bone (21.6%) was the most common metastatic site in patients with exon 20 insertions, followed by the central nervous system (CNS) (13.0%), liver (17.4%). Patients with intrathoracic metastases were more common in patients with exon 20 insertions (52.2%) than in those with major mutations (35.2%), although the differences were not significant. Of the 23 patients with exon 20 insertions, four were assessed for variants of exon 20 insertions by NGS**.**

**Table 1:** Characteristics of patients harboring EGFR exon 20 insertions and major mutations at diagnosis.

	Exon 20 insertions	EGFR major mutations			p-value^a^
	N = 23	All (N = 534)	Ex19 del (N = 285)	L858R (N = 249)	
**Age (year), median (range)**	60 (27–88)	66 (28–88)	65 (32–88)	68 (28–87)	
≥ 75, n (%)	3 (13.0)	125 (23.4)	57 (20.0)	68 (27.3)	0.017
< 75, n (%)	20 (87.0)	409 (76.6)	228 (80.0)	181 (72.7)	0.318
**Sex, n (%)**					
Female	18 (78.3)	338 (63.3)	173 (60.7)	165 (66.3)	0.233
Male	5 (21.7)	196 (36.7)	112 (39.3)	84 (33.7)	
**Histology, n (%)**					
Ad	22(95.7)	521 (97.6)	277 (97.2)	244 (98.0)	0.450
Others	1(4.3)	13 (2.4)	8 (2.8)	5 (2.0)	
**Smoking, n (%)**					
Never	15 (65.2)	313 (58.6)	152 (53.3)	161 (64.6)	0.847
Current/Former	8 (34.8)	218 (40.8)	132 (46.3)	86 (34.5)	
Unknown	0	3 (0.6)	1 (0.4)	2 (0.8)	
**Stage, n (%)**					
IVA/IVB recurrence	16 (69.6)	309 (57.9)	173 (60.7)	136 (54.6)	0.290
	7 (30.4)	225 (42.1)	112 (39.3)	113 (45.4)	
**Metastasis, n (%)**					
Bone	5 (21.6)	225 (42.1)	128 (44.9)	97 (39.0)	0.055
CNS	3 (13.0)	134 (25.1)	76 (26.7)	58 (23.3)	0.225
Liver	4 (17.4)	61 (11.4)	37 (13.0)	24 (9.6)	0.330
Intrathoracic disease	12 (52.2)	188 (35.2)	94 (33.0)	94 (37.8)	0.322

*Ad* Adenocarcinoma, *CNS* Central Nervous System.

^a^Comparison of EGFR exon 20 insertions and major mutations.

### Efficacy of platinum doublet chemotherapy in patients with exon 20 insertions

**Figure 1:**
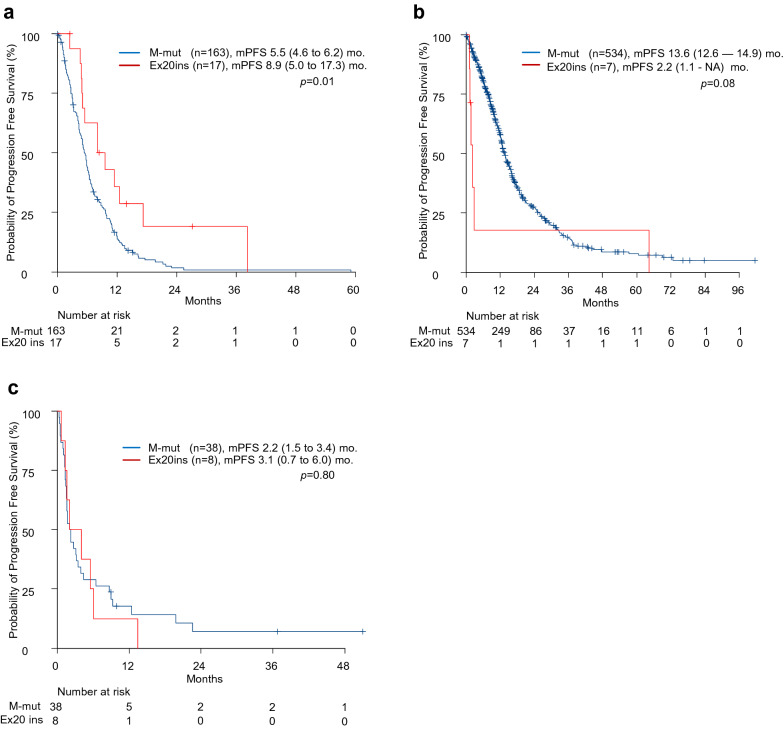
Median progression free survival after (**a**) platinum doublet chemotherapy, (**b**) EGFR-TKIs, and (**c**) anti-PD-1 antibody treatment in patients with EGFR exon 20 insertions and major mutations (L858R and exon 19 deletions).

Of the 23 patients with exon 20 insertions, 17 received platinum doublet chemotherapy, including two patients who received platinum doublet chemotherapy in combination with anti-PD-1 antibody, and 1 in combination with EGFR-TKIs. Other first-line treatments were as follows: four pembrolizumab, one EGFR-TKI, and one pemetrexed monotherapy (Supplementary Table 1). The ORR and mPFS of first-line platinum doublet chemotherapy in patients with exon 20 insertions were 11.8% (95% CI 1.5–36.4), and 8.9 months (95% confidence interval [CI] 5.0–17.3), compared with ORR of 21.5% (95% CI 15.4–28.6) and PFS of 5.5 months (95% CI 4.6–6.2) in patients with major mutations (ORR: *p* = 0.75; PFS: *p* = 0.01, Table 2 and Fig. 1a).

**Table 2:** Response of systemic therapy in patients with EGFR exon 20 insertions and major mutations.

Types of systemic therapy		PR	SD	PD	NE	ORR	*p*-value
Platinum doublet chemotherapy	Ex20ins^a^ (N = 17)	2	13	1	1	11.8% (1.5–36.4%)	0.75
	Major (N = 163)	35	82	41	5	21.5% (15.4–28.6%)	
EGFR-TKIs	Ex20ins (N = 7)	0	1	5	1	0% (0–3.5%)	0.003
	Major^a^ (N = 534)	309	148	34	43	57.9% (53.5–62.1%)	
Anti-PD-1 antibody	Ex20ins (N = 8)	2	2	4	0	25% (3.2–65.1%)	0.61
	Major (N = 38)	6	9	23	0	15.8% (6.0–31.3%)	

*PR* partial response, *SD* stable disease, *PD* progressive disease, *NE* non-evaluable, *EGFR-TKI* epidermal growth factor receptor-tyrosine kinase inhibitor, *Ex 20ins* exon 20 insertions, *PD-1* programmed cell death-1.

^a^First-line setting.

### Efficacy of EGFR-TKIs in patients with exon 20 insertions

Over the clinical course in patients with exon 20 insertions, 7 patients received EGFR-TKIs. The differences in the ORR and PFS between patients with exon 20 insertions and major mutation shown in Table 2 and Fig. 1b. The ORR and mPFS of EGFR-TKIs were 0%, 2.2 months (95% CI 1.1 to NA) in patients with exon 20 insertions and 57.9% (95% CI 53.5–62.1), 13.6 months (95% CI 12.6–14.9) in those with major mutation (ORR: *p* = 0.003 and, PFS: *p* = 0.08).

### Efficacy of anti-PD-1 antibody in patients with exon 20 insertions

Eight patients received anti-PD-1 antibody monotherapy in patients with exon 20 insertions. The differences in the ORR and PFS between exon 20 and major mutation patients in the anti-PD-1 antibody monotherapy are shown in Table 2 and Fig. 1c. ORR and PFS of anti-PD-1 antibody monotherapy was 25% (95% CI 3.2–65.1), 3.1 months (95% CI 0.7–6.0) in patients with exon 20 insertions, and 15.8% (95% CI 6.0–31.3), 2.2 months (95% CI 1.5–3.4) in those with major mutation (ORR: *p* = 0.61 and, PFS: *p* = 0.80).

### Overall survival in advanced NSCLC patients with exon 20 insertions

**Figure 2:**
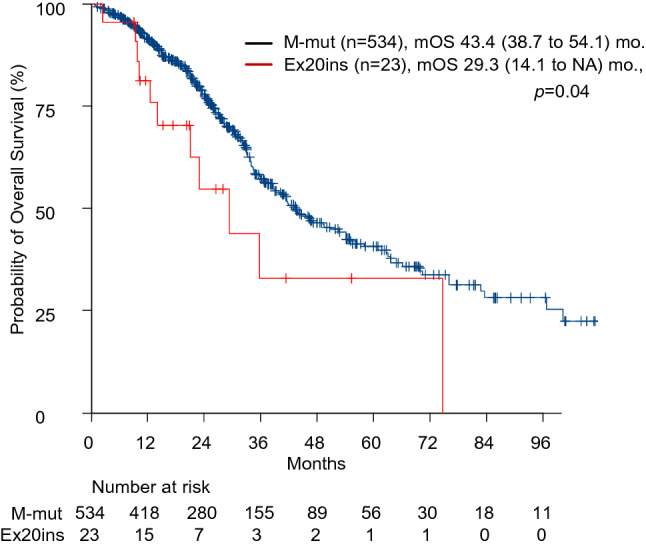
Overall survival in patients with EGFR exon 20 insertions and major mutations (L858R and exon 19 deletions).

The median overall survival in patients with exon 20 insertions was 29.3 months (95% confidence interval [CI] 14.1). On the other hand, OS in patients with major mutations who received EGFR-TKIs was 43.4 months (95% CI 38.7–54.2). Patients with exon 20 insertions had a significantly shorter OS than those with major mutations (*p* = 0.04, Fig. 2). The clinical outcomes of the four patients with the identified variants are shown in Supplementary Table 2.

## Discussion

We found that there were no significant differences in clinical characteristics, including the distribution of metastatic sites between patients with *EGFR* exon 20 insertion and major mutations. The OS of patients with exon 20 insertions was significantly shorter than in patients with major mutations who received EGFR-TKIs as initial treatment.

Few reports have focused on the differences in clinical characteristics between patients with exon 20 insertions and major mutations. Previous studies have shown that *EGFR* exon 20 insertion is more likely to occur in never or light smoking patients and those with lung adenocarcinomas^26,27^. In our study, however, there were no differences in sex, smoking history, histology, metastatic spread or stage at diagnosis between the two groups, while patients with exon 20 insertions were significantly younger than those with major mutations.

*EGFR* exon 20 insertions are related to the intrinsic resistance to conventional EGFR-TKIs compared with major mutations, such as exon 19 deletion and L858R in exon 21^4,12,28^. Due to the limited efficacy of EGFR-TKIs, platinum combination chemotherapy is still the standard therapy for patients with exon 20 insertion. Previous studies have reported that mPFS was 4.2–6.4 months and OS was 16.4–29.4 months, which were similar to our data^16,29,30^. On the other hands, the clinical efficacy of EGFR-TKIs in patients with *EGFR* exon 20 insertion has been reported to differ according to the variant^26^. Some variants such as EGFR A763_Y764insFQEA mutation have been reported to associate with sensitivity to first generation EGFR TKIs in both preclinical and clinical setting^26,31–33^. However, in the current clinical practice, we did not necessarily obtain detailed variant information, and the frequency of sensitive variants seems quite low. Thus, our results strongly support that *EGFR* exon 20 insertions are not sensitive to the conventional EGFR-TKIs.

We also evaluated the efficacy of anti-PD-1/PD-L1 antibodies in NSCLC with *EGFR* exon 20 insertions. In general, anti-PD-1/PD-L1 antibodies are poorly effective in *EGFR*-mutated NSCLC compared with those without *EGFR* mutations^34–36^. In this study, the ORR and mPFS of the anti-PD-1 antibody in patients with *EGFR* exon 20 insertions were 25% and 3.1 months (95% CI 0.7–6.0). Recent studies have reported that patients with *EGFR* exon 20 insertions showed better clinical outcomes of anti-PD-1 antibody compared with those with *EGFR* major mutations^37^. However, the therapeutic effect is still limited in patients with *EGFR* exon 20 insertions, and more specific treatment for advanced NSCLC with *EGFR* exon 20 insertions is desirable.

Recently, novel targeted therapies against *EGFR* exon 20 insertion mutations, such as poziotinib, mobocertinib, and amivantmab have been developed^17–20^. Poziotinib, a potent TKI against *EGFR* and *HER2* exon 20 insertion mutations, showed an ORR of 15–44% and PFS of 4.2–5.6 months in the phase II trial and results from the expanded access program^38–40^. Mobocertinib is an EGFR-TKI with potent and selective preclinical inhibitory activity against *EGFR* exon 20 insertions, with an ORR of 43% and PFS of 7.3 months in a phase II trial^41^. A phase III trial comparing mobocertinib with platinum-based chemotherapy as first-line therapy is currently ongoing (NCT04129502)^42^. Amivantamab is an anti-EGFR-MET bispecific antibody that can target diseases driven by both EGFR and MET, and has shown therapeutic efficacy in patients with a variety of mutations, including *EGFR* C797S, T790M, exon20 insertion mutation, and *MET* amplification. Amivantamab showed a response rate of 36% and a PFS of 8.3 months in a Phase II/III study^20^. A study is planned for advanced NSCLC patients with *EGFR* exon 20 insertion mutations, with carboplatin and pemetrexed with and without amivantamab (NCT04538664).

This study has some limitations. First, it is a single-center, retrospective study with a small sample size as patients with EGFR exon 20 mutations are rare. Additionally, genetic variants of exon 20 insertion were assessable in only four patients, as PCR-based testing showed only the presence of exon 20 insertion, not variant types, NGS was not approved for the detection of *EGFR* mutation at the testing time. *EGFR* exon 20 insertions are structurally and pharmacologically heterogeneous, with variability in their position and size having implications for response to conventional EGFR TKIs^43–45^. In this study, only four patients had detailed information on insertion variants, and we three different variants of EGFR exon 20 insertions. Indeed, preclinical studies showed A767_V769dupASV, A767_S768insTLA and D770_N771insSVD mutation which was similar variant to D770_N771insASV, are associated with resistance to first-generation EGFR TKIs, while showing a wide therapeutic window for osimertinib in preclinical studies^46,47^.

In conclusion, the OS of patients with exon 20 insertions was significantly shorter than those with major mutations due to the lack of targeted therapies, although clinical characteristics, including the distribution of metastatic sites was very similar between two groups. Additionally, the effectiveness of anti-PD-1 antibodies in patients with *EGFR* exon 20 insertion is limited as with those with EGFR major mutations. Therefore, the development of novel targeted therapies against NSCLC with *EGFR* exon 20 insertion mutations is warranted to improve the prognosis. On the other hands, EGFR exon 20 insertion is heterogeneous group of aberrations. Further investigation on association how the heterogeneous nature of EGFR exon 20 insertion mutations affect the clinical outcomes including the efficacy of these drugs will be warranted.

## Supplementary Information

Supplementary Tables.

## Data Availability

The datasets generated during and/or analysed during the current study are available from the corresponding author on reasonable request.
