# Supplementary material for: Clinical characteristics of advanced non-small cell lung cancer patients with EGFR exon 20 insertions
Source: Sci Rep. 2021 Sep 21;11:18762. doi: 10.1038/s41598-021-98275-3 (PMC8455549; doi:10.1038/s41598-021-98275-3)
Supplement: Supplementary file 1 — Supplementary Tables. [file 41598_2021_98275_MOESM1_ESM.docx]

**Supplemental Table 1: First-line treatments in patients with exon20 insertion mutation**

|  | patients, n(%) |
| --- | --- |
| **Platinum doublet(n=17)** |  |
| CDDP(CBDCA)+PEM | 9(39.1) |
| CBDCA+nab-PTX | 1(4.3) |
| CDDP(CBDCA)+PEM+BEV | 4(17.4) |
| CBDCA+PTX+BEV+Atezolizumab | 1(4.3) |
| CBDCA+PEM+Pembrolizumab | 1(4.3) |
| CBDCA+PEM+Gefitinib | 1(4.3) |
| **EGFR-TKIs(n=1)** |  |
| Erlotinib | 1(4.3) |
| **Anti-PD-1 monotherapy(n=4)** |  |
| Pembrolizumab | 4(17.4) |
| **Other(n=1)** |  |
| PEM | 1(4.3) |

CDDP, Cisplatin; CBDCA, Carboplatin; PEM, Pemetrexed; nab-PTX, nab-Paclitaxel; BEV, Bevacizumab.

**Supplemental Table 2. Clinical outcomes according to the variants of exon20 insertions**

| **No** | **sex** | **age** | **smoking** | **PS** | **stage** | **Variants of　Exon 20 insertion** | **EGFR-TKIs** | **Chemotherapy** | **Best Response** | **PFS**  **(month)** | **OS**  **(month)** |
| --- | --- | --- | --- | --- | --- | --- | --- | --- | --- | --- | --- |
| 1 | F | 52 | Never | 1 | IVA | A767_S768insTLA | N/A | CBDCA+PEM | SD | 17.3 | 18.3 |
| 2 | F | 58 | Never | 1 | IVB | A767_V769dupASV | N/A | CDDP+PEM | SD | 14.2 | 29.3 |
| 3 | F | 51 | Never | 0 | IVA | D770_771insASV | N/A | CDDP+PEM | SD | 12.5 | 39.7 |
| 4 | F | 54 | Never | 1 | IVA | A767_V769dupASV | N/A | CDDP+PEM | PD | 2.5 | 14.1 |

CDDP, cisplatin; CBDCA, carboplatin; PEM, pemetrexed; SD, stable disease; PD, progressive disease; N/A, not applicable.
